# *Cyclosa* Menge, 1866 (Araneidae) Orb‐Weavers Build Stabilimenta That Resemble Larger Spiders

**DOI:** 10.1002/ece3.72371

**Published:** 2025-11-06

**Authors:** George Olah, Phillip J. Torres, Aaron F. Pomerantz, Richard Kirby, Simon Baxter, Juan Grados, Lawrence E. Reeves

**Affiliations:** ^1^ Fenner School of Environment and Society The Australian National University Canberra ACT Australia; ^2^ Discovery Channel New York City New York USA; ^3^ Oxford Nanopore Technologies Alameda California USA; ^4^ Richard Kirby Photography Stroud UK; ^5^ BBC Natural History Unit Bristol UK; ^6^ Departamento de Entomología Museo de Historia Natural, UNMSM Lima Peru; ^7^ Florida Medical Entomology Laboratory, Institute of Food and Agricultural Sciences University of Florida Gainesville Florida USA

**Keywords:** defensa contra depredadores, Filipinas, Perú, señuelo de araña, decoy spider, Peru, Philippines, predator defense

## Abstract

Orb‐weaving spiders are known to create stabilimenta—silk situated at particular locations of the web. While anecdotal reports and popular media have long suggested that some spiders arrange debris in their webs to resemble a larger spider, this behavior has not been formally documented in the scientific literature. Here, we provide the first scientific record of this unique behavior in two orb‐weaving spiders (*Cyclosa* spp., Araneidae) from the tropical forests of Peru and the Philippines. We report that these spiders construct stabilimenta composed of detritus and silk, arranging the debris in the web into a shape that visually resembles the silhouette of a larger spider. This structure may serve as a “decoy” that serves an anti‐predator function of misdirecting or repelling the attacks of some predators. Video abstract: https://youtu.be/GDySHFRXbCE.

## Graphical abstract

The orb‐weaving spider *Cyclosa* *longicauda* from Peru constructs unique stabilimenta from detritus and silk that visually resemble a larger spider. This previously undescribed behavior likely functions as a sophisticated anti‐predator strategy, with the structure serving as a decoy to misdirect or repel attacks. The discovery of this complex behavior in two disparate locations (Peru and the Philippines) highlights a remarkable case of defensive web architecture. (Photographed by Richard Kirby).

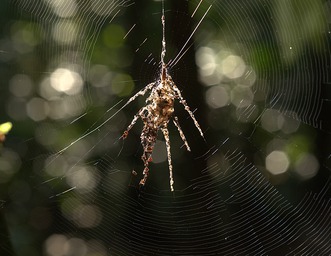

## RESUMEN

Las arañas tejedoras de orbes son conocidas por crear estabilimentos, estructuras de seda situadas en lugares específicos de la telaraña. Si bien los informes anecdóticos y los medios populares han sugerido durante mucho tiempo que algunas arañas organizan escombros en sus telas para parecerse a una araña más grande, este comportamiento no ha sido documentado formalmente en la literatura científica. En este trabajo, proporcionamos el primer registro científico de este comportamiento único en dos especies de arañas tejedoras de orbes (*Cyclosa* spp., Araneidae) de los bosques tropicales de Perú y Filipinas. Reportamos que estas arañas construyen estabilimentos compuestos con detritos y seda, organizando los escombros en la tela en una forma que se asemeja visualmente a la silueta de una araña más grande. Esta estructura puede servir como un “señuelo” que cumple una función anti‐depredador, desorientando o repeliendo los ataques de algunos depredadores. Video resumen: https://youtu.be/GDySHFRXbCE.

## 1 Introduction

Many diurnal orb‐weaving spiders (Araneidae family) build a silken retreat to hide from predators, while others build various web decorations (McCook 1889), also known as stabilimenta (Simon 1895), but rarely are both behaviors seen in the same species (Eberhard 1990). This suggests a fundamental trade‐off, where stabilimenta may serve a similar protective function as a physical retreat (Walter and Elgar 2012; Walter 2024). Stabilimenta typically consist of silk situated at particular positions on the web that are most often located at its hub (Herberstein et al. 2000). Despite considerable attention from researchers, the function of stabilimenta in Araneidae and other stabilimentum‐building spider families remains unclear (Bruce 2006; Walter 2024). A number of hypotheses have been proposed to explain the role of stabilimenta, including prevention of web collisions by birds (Eisner and Nowicki 1983; Bruce et al. 2005), prey attraction (Craig and Bernard 1990; Kim et al. 2012), and predator defense (Robinson and Robinson 1970; Soley 2019; Robledo‐Ospina et al. 2023).

The diverse (180 species globally; World Spider Catalog 2025), pan‐tropical araneid genus *Cyclosa* Menge, 1866, along with a monotypic genus, *Allocyclosa* Levi 1999, build stabilimenta that often incorporate masses of organic material, which are usually the carcasses of their arthropod prey or plant debris (Eberhard 2003; Marson 1947). Many species position egg sacs in cryptic locations on the stabilimentum (McClintock and Dodson 1999). The stabilimenta built by species in these genera are positioned toward the hub of the web and take on a variety of forms, but often debris is arranged in a discoid or linear shape (Herberstein et al. 2000). These stabilimenta are predominantly thought to function as a form of camouflage (Eberhard 2003; Tan and Li 2009; Liu et al. 2014), as the coloration of the spider often closely matches that of the stabilimentum. Concomitantly, the construction of stabilimenta gives *Cyclosa* webs a greater degree of conspicuousness to predators (Tseng and Tso 2009).

Studies on the response of visual‐hunting hymenopteran predators to various degrees of web decoration in two *Cyclosa* species found that spiders constructing larger stabilimenta suffer more predation attempts than undecorated webs, but predation attempts were less likely to be successful than those attacking undecorated webs (Chou et al. 2003; Tseng and Tso 2009). In these studies, hymenopteran attacks were directed toward the stabilimenta, suggesting that the *Cyclosa* stabilimentum serves to misdirect predation attempts as predators may have difficulty visually distinguishing the stabilimentum from the spider. Studies by Hoffmaster (1982) investigated the response of *Cyclosa caroli* (Hentz, 1850) and other araneids to predation attempts by hummingbirds and jumping spiders and found that predation attempts by hummingbirds were directed at the stabilimentum, largely unsuccessful, and elicited no response from the majority of *C. caroli* that were tested. In response to jumping spider predation attempts, most *C. caroli* responded with active defense movements, with some *C. caroli* dropping from the web. In contrast, none of the tested *C. caroli* left the web during attempted predation by the hummingbirds. There is some evidence that *Cyclosa* stabilimenta might promote prey attraction (Tso 1998; Tan et al. 2010) or serve to strengthen the web during adverse weather conditions (e.g., wind, rain) (Neet 1990).

Here, we present detailed field observations on the construction of complex stabilimenta by two geographically isolated *Cyclosa* species. While anecdotal accounts have previously suggested that some spiders arrange debris in their webs to resemble a larger spider, this behavior has not been formally documented in the scientific literature. Our study aims to fill this gap by providing the first comprehensive record of this intriguing behavior, describing the step‐by‐step construction of these decorations and the sequence of the web‐building process. As far as we are aware, the only other scientific records similar to these are from populations of *Cyclosa fililineata* Hingston, 1932 and *Cyclosa morretes* Levi 1999 in southeastern Brazil, with a more rudimentary stabilimentum described as a blob with extensions resembling legs. These forms made up less than 20% of the studied populations (Gonzaga and Vasconcellos‐Neto 2012), highlighting the much more complex and frequent behavior we report here.

## 2 Materials and Methods

Field observations were made on species of *Cyclosa* in Peru in 2012, 2014, and 2022, and in the Philippines in 2012. The Peruvian *Cyclosa* were observed near the Tambopata Research Center (TRC) in the Tambopata National Reserve, Madre de Dios, in the Southeastern Peruvian Amazon. The habitat was a lowland tropical moist forest with an average elevation of 250 m and annual rainfall of about 3300 mm (Brightsmith 2004). The initial observations were made in 2012. More detailed observations were documented in 2014 and 2022. In May 2014 (beginning of the region's annual dry season), observations were made on 48 individual *Cyclosa* spiders in the Tambopata National Reserve, located by visually surveying the undergrowth along trails at the TRC. When *Cyclosa* webs were found, they were photographed using a Canon 7D digital camera with a Canon 100 Mm L macro lens. For even scaling, we obtained measurements of each spider's body and stabilimentum by maintaining a constant focusing distance and aperture on the camera lens and taking a second photograph of a metric ruler. This approach was used to avoid disruption of the web or spider that may have occurred if a ruler had been held against the web. Using this method, we measured the length of each spider's abdomen and the width of each stabilimentum at its widest point. Measurements were made by combining the two images in Adobe Photoshop CS6 Version 13.0. Each spider was then given a unique identifying number. The approximate location of each web was marked with fluorescent pink flagging.

*Cyclosa* spiders were again observed between 19 September and 09 October 2022 (end of the region's annual dry season). During the second expedition, we identified around 20 *Cyclosa* webs near the TRC, which we then monitored every day, returning at least once within six hours to each web, and conducting non‐stop observation on selected webs between 22.00 and 06.00 local time. The study was conducted with permission of the Servicio Nacional de Áreas Naturales Protegidas (SERNANP, N^o^ 99‐2022‐SERNANP‐JRNTAMB). Photos and videos were taken of specimens with different “spider‐like stabilimenta”, putative decoys, in their web. We collected nine specimens and placed them into uniquely labeled Eppendorf tubes with 80% ethanol. Collected specimens were sent to Lima, Peru and identified by Dr. D. Silva under light microscopy at the Peruvian Natural History Museum, based on their morphology and epigynum (Levi 1999), while comparing to vouchered specimens in the museum's collection.

In the Philippines, *Cyclosa* observations were made in Negros Occidental on the Island of Negros, Visayan Region, in a secondary tract of sub‐montane rainforest on the outside perimeter of Mount Kanlaon Natural Park, near the municipality of Murcia, at an approximate elevation of 625 m. observations were made during the course of fieldwork studying butterflies in Mount Kanlaon Natural Park (Reeves and Daniels 2020) in March 2012, late in the region's annual dry season. Under this work, we were not permitted to collect spider specimens. Thus, we were unable to observe the morphology of the spiders in the laboratory to determine the species engaging in this behavior. Six *Cyclosa* spiders with stabilimenta consisting of a central mass of debris with attenuate linear projections radiating outwards were first noticed on 8 March 2012 at 16.45 local time, when spiders and their webs were documented with photographs and fieldnotes. We photographed each individual and their webs using a Canon 7D digital camera with either a Canon 100 Mm macro lens or a Canon MPE 65 m macro lens. Some of the observed spiders were located adjacent to trails that were used to access field sites in Mount Kanlaon Natural Park, and these individuals were seen with their stabilimenta on multiple occasions in March 2012 in passing but were not photographed or observed in detail. Digital images of *Cyclosa* from the Philippines and Peru are preserved in the photograph archives of L. E. Reeves, P. J. Torres, and R. Kirby.

## 3 Results

Both the Peruvian and Philippine species were observed in orb webs containing stabilimenta made of prey carcasses and plant detritus. In both locations, the stabilimenta contained a central mass of debris at the hub of the web, with a number of linear projections radiating outwards from the central mass.

**FIGURE 1:**
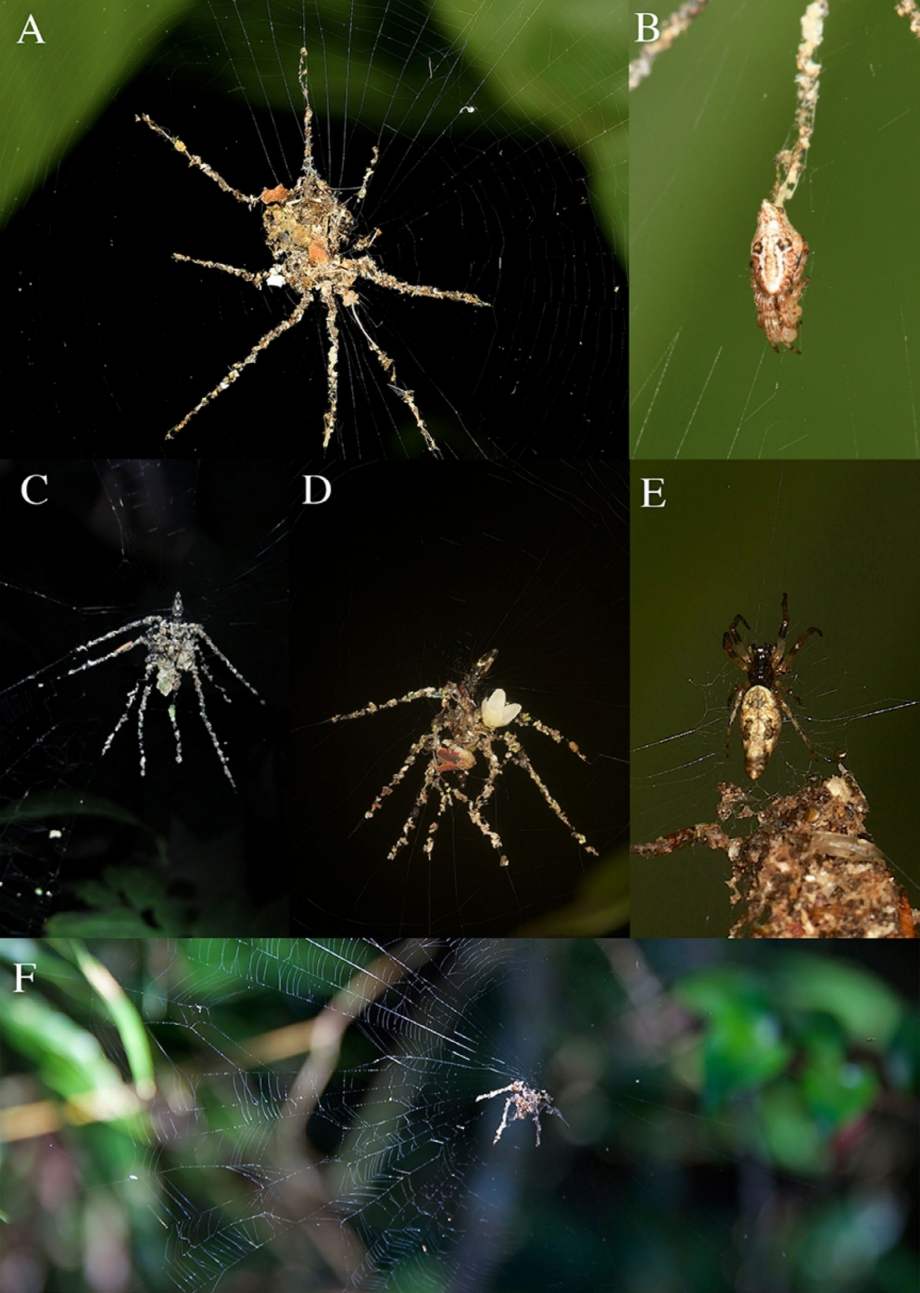
(A) The “spider‐like decoy” stabilimentum of the first individual *Cyclosa* sp. observed in the Philippines. (B) The *Cyclosa* sp. observed resting within the stabilimentum illustrated in Figure 1A. (C) The first *Cyclosa* individual found exhibiting a spider‐like stabilimentum in Madre de Dios, Peru. (D) The stabilimentum and spider of the Peruvian *Cyclosa* sp. showing the spider's position above the stabilimentum. (E) The Peruvian *Cyclosa* species that builds stabilimentum. (F) A similar stabilimentum, built by an unknown spider taxon, found and photographed in the Torotorofotsy‐Ihofa forest, Madagascar by H. Cordey.

At the Philippine site, a single individual was initially observed in a web. The web was situated approximately 1 m from the ground in a coffee tree (*Coffea* sp.) immediately adjacent to a trail. The stabilimentum of the first individual observed had eight radial projections (Figure 1A). Altogether, six individuals were observed with similar stabilimenta, presumably the same species. Among them, the number of radial projections varied from five to eight. The central mass at the hub of the web was constructed as a shallow envelope or pocket, and all individual spiders were observed resting within. All webs were found along an approximately 20 m stretch of the trail. Despite extensive work in the vicinity over a three‐month period, this section of trail was the only area where *Cyclosa* species were found.

In Peru, we observed *Cyclosa* spiders at the TRC with stabilimenta consisting of a central mass at the hub of the web with radial projections. The first stabilimentum seen (Figure 1C) was initially mistaken as a spider killed by an entomophagous fungus and was documented in September 2012. After further inspection, three individuals, presumably of the same species, were found at this site occupying similar webs with spider‐like stabilimenta, all 1–2 m from the ground and located within 2 m of each other. Between 2013 and 2024, it is estimated that over 250 individuals have been seen by eco‐guides and naturalists at TRC exhibiting similar stabilimenta. In contrast to the Philippine *Cyclosa*, the central mass of the stabilimentum was located directly below the hub, on which the spider was resting. The projections extended radially from the hub and all pointed in the same direction, most often downwards, and varied in number from four to eight. These spiders were only observed in floodplain forest and were absent in the higher *terra firme* forest nearby.

Of the 48 *Cyclosa* spiders photographically measured in 2014, the mean abdomen length was 2.54 mm, and the mean width of the central mass of the stabilimentum was 8.15 mm (excluding legs). On average, the length of the stabilimenta was 3.3 times that of the spider's body length. Among these spiders, the mean number of stabilimentum projections was 5.02. Of the spiders we photographed, we found both males and females to be occupying stabilimentum‐adorned webs. Some females had camouflaged egg sacs and occasionally, spiderlings, hidden among the debris of the stabilimentum. In 2014, we observed that some of the *Cyclosa* spiders (later identified as different species) with stabilimenta that consisted of a single line were observed in sympatry with those creating the more complex stabilimenta, but notes on these individuals were not recorded.

In 2022, we encountered around 20 *Cyclosa* spiders at the same floodplain area of the TRC. Some decorated their web with a single‐line stabilimentum, while others built the more complex structure. When the latter were approached by human observers, either during the day or at night with a flashlight, they started to shake their abdomens against the stabilimentum, making a vibration effect on the putative spider model. When further approached, the spider jumped off from the web to the ground (a height of about 1 m), but after an hour, it reappeared in its web. We did not observe such shaking behavior from spiders with a single‐line stabilimentum.

In 2022, observations were made on the maintenance of *Cyclosa* webs and stabilimenta. After sunset, between 18.00 and 22.00 local time, the spiders collected detritus from the spiral threads of their webs to the radials. Then, they ate the segments of the web and pushed the detritus along the radial spoke to the central hub, which happened very quickly. Later, around 02.00, the spiders continued to build their web by arranging the detritus over the main radials (1/4 of the final radials) resembling “legs” of the stabilimentum. Noticeably, as the spiders collected detritus, they would frequently reject some material, cutting it out of the web and flicking it away with one leg. On observation, it seemed to be the larger pieces of detritus that were rejected, but no measurements were made. This “reject‐and‐flick” behavior was also seen when the spiders were caught in the rain. They would collect small beads of water from their bodies and flick them away with one of their front legs. Later, they added more radials and made an outward sparse spiral. Spiders spent a lot of time chewing through the larger detritus, while separating larger pieces of it into multiple parts and pulling them apart. Finally, they made a very tight and fine inward spiral. On average (*n* = 6), it took them about 90 min to build a complete stabilimentum. We noticed that web building appeared to be a two‐stage process wherein the spiders would build the basic structure of the web around 23.00–24.00 after moving to where they wanted to build. Then, they rested for a few hours before spinning the tight spirals an hour or two before dawn (~06.00).

**FIGURE 2:**
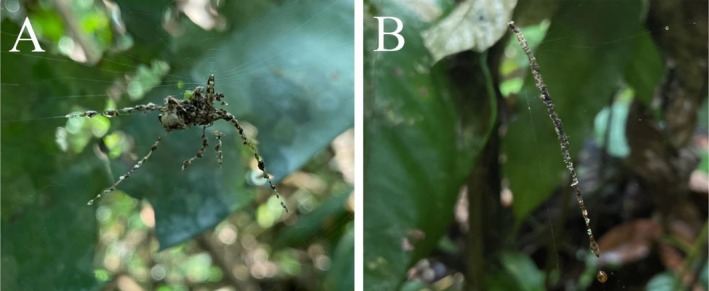
Stabilimenta of *Cyclosa* spiders identified in Tambopata, Peru. (A) *Cyclosa longicauda* (Taczanowski, 1878), with a “spider‐shaped” model. (B) *Cyclosa inca* Levi 1999, with a single‐line stabilimentum.

Though we could not collect specimens of the Philippine species for identification, we were able to identify the nine specimens collected at the TRC to species level. Five specimens (two females and three juveniles) that constructed the complex, spider‐like stabilimenta were *Cyclosa longicauda* (Taczanowski, 1878) (Figure 2A). The other four specimens (one female and three juveniles) constructing single‐line stabilimenta belonged to the species *Cyclosa inca* Levi 1999 (Figure 2B).

## 4 Discussion

Unlike many orb‐weaving spiders that construct a silken retreat for protection, the *Cyclosa* species we observed typically do not. This mutually exclusive behavior suggests that web stabilimenta and physical retreats serve a similar purpose: providing protection from predators. The diverse, pan‐tropical genus *Cyclosa* is known to incorporate plant detritus, the remains of prey, and other organic material into a stabilimentum at the hub of its web. The arrangement of organic matter can vary in *Cyclosa* stabilimenta. Most often, *Cyclosa* species arrange organic material or silk into a linear shape. Here, we provide the first formal scientific record of *Cyclosa* species from two continents that create stabilimenta visually resembling the shape of a larger spider.

In Peru, the spiders constructing spider‐like stabilimenta were morphologically identified as *C. longicauda* , while those constructing linear stabilimenta were identified as *C. inca* . *Cyclosa longicauda* was described in 1878 from a subadult female collected in Junín Department, Peru (Taczanowski 1878). The species is not well known, and the few records of the species are distributed from the upper Amazon of Colombia and Ecuador, through Peru and Brazil, to Misiones Province of Argentina (Levi 1999). The holotype of *C. inca* was collected by D. Silva in 1987 near Huánuco, Peru, and it has been collected since by fogging canopy in Tambopata, Peru, and in Colombia (Levi 1999). The Natural History Museum (National University of San Marcos) in Lima houses specimens of two additional *Cyclosa* species previously collected from the Tambopata region, which are yet to be identified (D. Silva, pers. comm.).

The incorporation of prey carcasses and plant debris into the stabilimenta of *Cyclosa* species is generally regarded as a defense against predation that serves to redirect the attacks of visually hunting predators and camouflage the spider (Eberhard 2003; Neet 1990; Tan and Li 2009). In both Neotropical and Southeast Asian forests, small araneids like *Cyclosa* are under predation pressure from a suite of visual diurnal predators. In the Neotropics and Southeast Asia, hummingbirds and nectariniids (sunbirds and spiderhunters), respectively, are known to glean orb‐weaving spiders from their webs (Skutch 1964; Stiles 1995). During a predation attempt, it is likely that concealment inside its spider‐like stabilimentum by the Philippine species would be beneficial to the spider by allowing it to escape detection until the predator abandons its attack. The Peruvian species, on the other hand, perches on the web directly above its spider‐like stabilimentum and, to the human eye, appears as a small projection relative to the larger structure. The spider may be attempting to direct potential predators toward the stabilimentum and away from itself, escaping by abandoning the web in the event of a predation attempt.

Helicopter damselflies (Odonata: Pseudostigmatidae) are specialist predators of web‐building spiders, and they occur extensively throughout the Neotropics, including the region where the Peruvian *C. longicauda* are found. At the TRC, we confirmed the presence of the pseudostigmatid species *Mecistogaster linearis* (Fabricius, 1777) and *Microstigma rotundatum* Selys, 1860 in the same habitat as *C. longicauda* . Fincke (1992) described pseudostigmatids as visual predators that will methodically search vegetation for spider webs. When a web is located, the damselfly hovers in front of the web searching for the spider. Pseudostigmatids have been reported to target araneid spiders, including *Cyclosa*, that are 3–6 mm in body length, while actively avoiding larger species (Fincke 1992). Perhaps *C. longicauda* evolved to construct these putative decoys in response to predation pressure by pseudostigmatid damselflies. Based on the measurements we collected on the Peruvian *C. longicauda*, this spider is within the preferred size range of damselflies, while the spider‐like stabilimentum that it constructs is within the size range of a spider that would be avoided. On the other hand, this form of deception might be even more effective against vertebrate predators, such as birds or lizards, which, unlike insects, are known to respond strongly to macroscopic patterns and may be confused by the large, spider‐like decoy, as reported about *Argiope* spiders (Schoener and Spiller 1992). Thus, the decoy could serve as a general defense against a suite of visually hunting predators.

On the other hand, the structures could also be interpreted as a form of bird dropping mimicry. This alternative hypothesis, as proposed by Liu et al. (2014) for *Cyclosa ginnaga* Yaginuma, 1959, suggests that the spiders' stabilimenta may resemble bird feces to avoid predation. The predominantly downward orientation of the stabilimentum's appendages in our observations (Figure 1C) could be interpreted as mimicking the irregular shape of a bird dropping. This would be a particularly effective defense against vertebrate predators like birds, as few would attempt to attack or consume their own waste (but see Oláh and Rózsa 2006). This suggests the decoy's function may be multifaceted, serving as a deterrent through both mimicry of a larger spider and an undesirable object.

A comparison between the different types of stabilimenta and the species that build them reveals potential functional differences. Linear stabilimenta are common and appear to be primarily for camouflage and attracting prey, while the “spider‐like” decoys we observed seem to have a more specific function of mimicking a larger, non‐prey spider. The construction of these complex decoys may require a greater energetic investment in collecting and arranging debris, but this could be offset by a higher survival rate against specific predators. In contrast, linear stabilimenta, which are often made of silk and smaller amounts of debris, might be less costly to produce. These differing investments may reflect distinct lifestyles between the two *Cyclosa* species identified in Peru. The species that creates the complex, spider‐like decoys may be more sedentary, as a higher investment in a single, long‐term defensive structure would be beneficial for a stationary lifestyle. In contrast, species that build less complex, linear decorations may relocate their webs more frequently, making a less effective but quickly constructed defense a more practical compromise. While our study did not directly investigate these lifestyle differences, our observations suggest that the different patterns of the stabilimenta may be linked to varying life histories, predator communities, and habitat characteristics.

Other genera, such as *Argiope*, construct elaborate stabilimenta that are often hypothesized to deter predators by increasing the apparent size of the spider (Hingston 1927). Our observations suggest a similar anti‐predator function, but through mimicry rather than simple enlargement. The decoys created by our *Cyclosa* species are not just larger, but they are shaped to resemble a potential threat or an undesirable prey item, potentially providing a more effective deterrent against predators like the damselflies we discussed earlier.

The two‐stage, sequential nature of web construction observed in our study (in which the spider‐like stabilimentum is built first at night, followed by the rest of the web closer to dawn) presents an interesting behavioral strategy. We hypothesize that this timing is a direct response to predation pressure. Given that the spiders' diurnal predators, such as hummingbirds and damselflies, are visually oriented, the spiders may prioritize creating their primary anti‐predator defense, the decoy, under the cover of darkness. This strategy allows them to complete the most critical part of their defense when visual predators are not active. The spiders can then rest and complete the finer parts of the web in the pre‐dawn hours, ensuring that their snare is ready for prey capture at first light. This would minimize the time the spiders are exposed without their primary defense during daylight hours. This sequential building process suggests that the stabilimentum is not just an incidental part of the web but an evolutionarily optimized and critical component of the survival strategy of this species.

This report summarizes field observations on the construction of putative decoy stabilimenta by *Cyclosa* species in the forests of Peru and the Philippines, a previously unreported behavior in spiders. In addition to these records, another spider, likely a species of *Cyclosa*, was observed by crew members of a nature documentary to create a spider‐like stabilimentum in the Torotorofotsy‐Ihofa Forest in eastern Madagascar in April of 2014 (Figure 1F). Taken together, the observations of this behavior in Peru, the Philippines, and Madagascar suggest that this behavior is widespread in *Cyclosa*. Considering the diversity of *Cyclosa* (180 described species) and its pan‐tropical distribution, it remains an open question what specific selective pressures have driven these particular populations or species to evolve such complex, unique “spider‐like decoys” while other species in the genus have maintained different “decorative” patterns or none at all. Perhaps there is particularly strong selection from predation within both of these groups that has resulted in increasingly complex visual defense. Future experimental studies could shed further light on this issue. For instance, comparing the survival rates of spiders with and without stabilimenta, or manipulating decoration size, could provide a more direct test of the protective function of these stabilimenta. Further investigation is necessary to determine the identity of the Philippine species described here and to elaborate on the function of their stabilimenta.

## Author Contributions

**George Olah:** conceptualization (equal), data curation (equal), formal analysis (equal), funding acquisition (equal), investigation (equal), methodology (equal), project administration (equal), resources (equal), visualization (equal), writing – original draft (equal), writing – review and editing (equal). **Phillip J. Torres:** conceptualization (equal), data curation (equal), investigation (equal), visualization (equal), writing – review and editing (equal). **Aaron F. Pomerantz:** investigation (equal), writing – review and editing (equal). **Richard Kirby:** investigation (equal), writing – review and editing (equal). **Simon Baxter:** investigation (equal), writing – review and editing (equal). **Juan Grados:** formal analysis (equal), investigation (equal), writing – review and editing (equal). **Lawrence E. Reeves:** conceptualization (equal), data curation (equal), formal analysis (equal), investigation (equal), methodology (equal), resources (equal), supervision (equal), validation (equal), visualization (equal), writing – original draft (equal), writing – review and editing (equal).

## Conflicts of Interest

The authors declare no conflicts of interest.

## Data Availability

This manuscript provides descriptive information, and all data are provided in included figures.

## References

[ece372371-bib-0001] Brightsmith, D. J. 2004. “Effects of Weather on Parrot Geophagy in Tambopata, Peru.” Wilson Bulletin 116: 134–145.

[ece372371-bib-0002] Bruce, M. J. 2006. “Silk Decoration: Controversy and Consensus.” Journal of Zoology 269: 89–97.

[ece372371-bib-0003] Bruce, M. J. , A. M. Heiling , and M. E. Herberstein . 2005. “Spider Signals: Are Web Decorations Visible to Birds and Bees?” Biology Letters 1: 299–302. 17148192 10.1098/rsbl.2005.0307 PMC1617156

[ece372371-bib-0004] Chou, I. , P. Wang , P. Shen , and I. Tso . 2003. “A Test of Prey‐Attracting and Predator Defence Functions of Prey Carcass Decorations Built by Cyclosa Spiders.” Animal Behaviour 69: 1055–1061.

[ece372371-bib-0005] Craig, C. L. , and G. D. Bernard . 1990. “Insect Attraction to Ultraviolet‐Reflecting Spider Webs and Web Decoration.” Ecology 71: 616–623.

[ece372371-bib-0006] Eberhard, W. G. 1990. “Function and Phylogeny of Spider Webs.” Annual Review of Ecology and Systematics 21: 341–372.

[ece372371-bib-0007] Eberhard, W. G. 2003. “Substitution of Silk Stabilimenta for Egg Sacs by *Allocyclosa bifurca* (Aranaea: Araneidae) Suggests Silk Stabilimenta Function as Camouflage Devices.” Behaviour 140: 847–868.

[ece372371-bib-0008] Eisner, T. , and S. Nowicki . 1983. “Spider Web Protection Through Visual Advertisement: Role of the Stabilimentum.” Science 219: 185–187. 17841687 10.1126/science.219.4581.185

[ece372371-bib-0009] Fincke, O. 1992. “Behavioural Ecology of the Giant Damselflies of Barro Colorado Island, Panama (Odonata: Zygoptera: Pseudostigmatidae).” In Insects of Panama and Mesoamerica, edited by D. Quintero and A. Aiello , 102–113. Oxford University Press.

[ece372371-bib-0010] Gonzaga, M. O. , and J. Vasconcellos‐Neto . 2012. “Variation in the Stabilimenta of *Cyclosa Fililineata* Hingston, 1932, and *Cyclosa morretes* Levi, 1999 (Araneae: Araneidae), in Southeastern Brazil.” Psyche (Cambridge) 2012: 1–10.

[ece372371-bib-0011] Herberstein, M. E. , C. L. Craig , J. A. Coddington , and M. A. Elgar . 2000. “The Functional Significance of Silk Decorations of Orb‐Web Spiders: A Critical Review of the Empirical Evidence.” Biological Reviews of the Cambridge Philosophical Society 75: 649–669. 11117202 10.1111/j.1469-185x.2000.tb00056.x

[ece372371-bib-0012] Hingston, R. W. G. 1927. “Protective Devices in Spiders' Snares, With a Description of Seven New Species of Orb–Weaving Spiders.” Proceedings of the Zoological Society of London 97, no. 2: 259–293.

[ece372371-bib-0013] Hoffmaster, D. K. 1982. “Predator Avoidance Behaviors of Five Species of Panamanian Orb‐Weaving Spiders (Aranaea: Araneidae, Uluboridae).” Journal of Arachnology 10: 69–71.

[ece372371-bib-0014] Kim, K. W. , K. Kim , and J. C. Choe . 2012. “Functional Values of Stabilimenta in a Wasp Spider, *Argiope bruennichi* : Support for the Prey‐Attraction Hypothesis.” Behavioral Ecology and Sociobiology 66, no. 12: 1569–1576.

[ece372371-bib-0015] Levi, H. W. 1999. “The Neotropical and Mexican Orb Weavers of the Genera *Cyclosa* and *Allocyclosa* (Araneae: Araneidae).” Bulletin of the Museum of Comparative Zoology at Harvard College 155: 299–379.

[ece372371-bib-0016] Liu, M. H. , S. J. Blamires , C. P. Liao , and M. N. Tso . 2014. “Evidence of Bird Dropping Masquerading by a Spider to Avoid Predators.” Scientific Reports 4: 5058. 24875182 10.1038/srep05058 PMC4038025

[ece372371-bib-0017] Marson, J. E. 1947. “Some Observation on the Variations in the Camouflage Devices Used by *Cyclosa insulana* (Costa).” Proceedings of the Zoological Society of London 117: 598–605.

[ece372371-bib-0018] McClintock, W. J. , and G. N. Dodson . 1999. “Notes on *Cyclosa insulana* (Araneae, Araneidae) of Papua New Guinea.” Journal of Arachnology 27: 685–688.

[ece372371-bib-0019] McCook, H. C. 1889. American Spiders and Their Spinning Work: Snares and Nests (Vol. 1).

[ece372371-bib-0020] Neet, C. R. 1990. “Function and Structural Variability of the Stabilimenta of *Cyclosa insulana* (Costa) (Araneae, Araneidae).” Bulletin of the British Arachnological Society 8: 161–164.

[ece372371-bib-0021] Oláh, G. , and L. Rózsa . 2006. “Nitrogen Metabolic Wastes Do not Influence Drinking Water Preference in Feral Pigeons.” Acta Zoologica Academiae Scientiarum Hungaricae 52, no. 4: 401–406.

[ece372371-bib-0022] Reeves, L. E. , and J. C. Daniels . 2020. “Conservation Value of Secondary Forest Habitats to Endemic Frugivorous Butterflies at Mount Kanlaon, Negros Occidental, Philippines.” Journal of Insect Conservation 24: 913–926.

[ece372371-bib-0023] Robinson, M. H. , and B. Robinson . 1970. “The Stabilimentum of the Orb Web Spider, *Argiope argentata* : An Improbable Defense Against Predators.” Canadian Entomologist 102: 641–655.

[ece372371-bib-0024] Robledo‐Ospina, L. E. , N. Morehouse , F. Escobar , H. Tapia‐McClung , A. Narendra , and D. Rao . 2023. “Visual Antipredator Effects of Web Flexing in an Orb Web Spider, With Special Reference to Web Decorations.” Science of Nature 110, no. 3: 23. 10.1007/s00114-023-01849-6 37219696

[ece372371-bib-0025] Schoener, T. W. , and D. A. Spiller . 1992. “Stabilimenta Characteristics of the Spider *Argiope argentata* on Small Islands: Support of the Predator‐Defense Hypothesis.” Behavioral Ecology and Sociobiology 31, no. 5: 309–318.

[ece372371-bib-0026] Simon, E. 1895. “Arachnides Recueillis par MG Potanine en Chinie et en Mongolie (1876–1879).” Bulletin de l'Académie impériale des sciences de St.‐Pétersbourg 5, no. 2: 331–345.

[ece372371-bib-0027] Skutch, A. F. 1964. “Life Histories of the Hermit Hummingbirds.” Auk 93: 5–25.

[ece372371-bib-0028] Soley, F. G. 2019. “A Possible Role of Decorations in Spiderwebs as Protection Devices That Distract Predators.” Revista de Biología Tropical 67, no. 2: 164–173.

[ece372371-bib-0029] Stiles, F. G. 1995. “Behavioral, Ecological and Morphological Correlates of Foraging for Arthropods by the Hummingbirds of a Tropical Wet Forest.” Condor 97: 853–878.

[ece372371-bib-0030] Taczanowski, L. 1878. “Les Aranéides du Pérou Central.” Horae Societatis Entomologicae Rossicae 14: 140–175.

[ece372371-bib-0031] Tan, E. J. , and D. Li . 2009. “Detritus Decoration of an Orb‐Weaving Spider *Cyclosa mulmeinensis* (Thorell): For Food or Camouflage?” Journal of Experimental Biology 212: 1832–1839. 19483001 10.1242/jeb.030502

[ece372371-bib-0032] Tan, E. J. , W. K. Seah , L. Y. L. Yap , P. M. Gan , F. Liu , and D. Li . 2010. “Why Do Orb‐Weaving Spiders (*Cyclosa ginnaga*) Decorate Their Webs With Silk Spirals and Plant Detritus?” Animal Behaviour 79: 179–186.

[ece372371-bib-0033] Tseng, L. , and I. Tso . 2009. “A Risky Defence by a Spider Using Conspicuous Decoys Resembling Itself in Appearance.” Animal Behaviour 78: 425–431.

[ece372371-bib-0034] Tso, I. 1998. “Stabilimentum‐Decorated Webs Spun by *Cyclosa conica* (Araneae, Araneidae) Trapped More Insects Than Undecorated Webs.” Journal of Arachnology 26: 101–105.

[ece372371-bib-0035] Walter, A. 2024. “The Function of Web Decorations in Orb Web Spiders.” Frontiers in Arachnid Science 3: 1384128.

[ece372371-bib-0036] Walter, A. , and M. A. Elgar . 2012. “The Evolution of Novel Animal Signals: Silk Decorations as a Model System.” Biological Reviews 87, no. 3: 686–700. 22309051 10.1111/j.1469-185X.2012.00219.x

[ece372371-bib-0037] World Spider Catalog . 2025. World Spider Catalog. Version 26. Natural History Museum Bern. https://wsc.nmbe.ch.10.24436/2.

